# “Languaging” tacit judgment in formal postgraduate assessment: the documentation of ad hoc and summative entrustment decisions

**DOI:** 10.1007/s40037-020-00616-x

**Published:** 2020-09-15

**Authors:** Anneke van Enk, Olle ten Cate

**Affiliations:** 1 Centre for Health Education Scholarship, University of British Columbia, Vancouver, Canada; 2 Centre for Research and Development of Education, University Medical Centre Utrecht, Utrecht, The Netherlands

**Keywords:** Entrustment, Tacit judgment, Assessment, Language

## Abstract

While subjective judgment is recognized by the health professions education literature as important to assessment, it remains difficult to carve out a formally recognized role in assessment practices for personal experiences, gestalts, and gut feelings. Assessment tends to rely on documentary artefacts—like the forms, standards, and policies brought in under competency-based medical education, for example—to support accountability and fairness. But judgment is often tacit in nature and can be more challenging to surface in explicit (and particularly written) form. What is needed is a nuanced approach to the incorporation of judgment in assessment such that it is neither in danger of being suppressed by an overly rigorous insistence on documentation nor uncritically sanctioned by the defense that it resides in a black box and that we must simply trust the expertise of assessors. The concept of entrustment represents an attempt to effect such a balance within current competency frameworks by surfacing judgments about the degree of supervision learners need to care safely for patients. While there is relatively little published data about its implementation as yet, one readily manifest variation in the uptake of entrustment relates to the distinction between ad hoc and summative forms. The ways in which these forms are languaged, together with their intended purposes and guidelines for their use, point to directions for more focused empirical inquiry that can inform current and future uptake of entrustment in competency-based medical education and the responsible and meaningful inclusion of judgment in assessment more generally.

Personal judgment, long regarded as undesirable noise in psychometrics, has recently been reframed as necessary and valuable in evaluating learner performance in complex and dynamic practice settings [1–4]. Yet the problem of how best to incorporate judgment in formal assessment in meaningful and defensible ways remains. For, while attempts at exclusively “objective” measurement have been missing the mark (and, worse, have at times contributed to systemic unfairness[5]), professional judgment is also subject to blind spots and, when not guided by and answerable to other stakeholders in assessment, can lend itself to confusion, arbitrariness and discrimination. To help guard against this, formal assessment employs various linguistic artefacts, including standards, scoring rubrics, procedural guidelines, evaluation forms, and more^1^. These artefacts spell out expectations, record achievement, and publish assessment policies and procedures, and they are generally considered essential to good assessment in higher education.

Competency-based medical education (CBME) is promoted as meeting the need for exactly such documented explicitness, for the practice of medicine at a “defined level of proficiency” [6]. In demanding formally reported evaluation on the basis of observable behaviours matched to specified, broadly circulated standards, CBME attempts to address concerns about a lack of clarity, consistency and fairness for learners [7] and to enhance accountability to the wider public [8]. Yet it may risk doing this at the cost of assessors’ more holistic judgments, which are often harder to surface in language [4, 9]. An otherwise legitimate requirement for explicitness in assessment can be taken so far as to imply that if assessors cannot completely justify their evaluations in accordance with published standards and on the basis of documented observation, then their assessment cannot count. Such a rigorous approach to explicitness rests, as educational philosophers Arnal and Burwood [10] point out, on “a fallacy: if you cannot tell, then you do not know.” The belief that a valid assessment can be fully captured in words—specifically, in sharply-defined and stable written text—is belied by the fact that, in the process of working with and teaching learners in clinics and operating rooms, assessors come to know far more about their capabilities than they are able (or, sometimes, willing) to write down. Consider, for instance, this comment from a report on the database multisourcefeedback.nl [11, 12] : “Sometimes it is difficult to derive from the EHR [electronic health record] entries what her proposed management policy is. But this does not mean that I think X doesn’t have it well in her mind; on the contrary.” Here a supervising clinician struggles to square a readily documentable observation about a learner with what they “just know” about her on the basis of practical experience. This subsidiary or background knowledge—which tends to operate implicitly and can be difficult to surface in language—allows assessors to intelligently interpret and act on the important but necessarily limited externalization presented by linguistic artefacts like standards, forms and policies.

## Entrustment requires a holistic judgment

The concept of entrustment in medical education can be understood as an attempt to capture what assessors know beyond CBME’s lists of specifications. Indeed, in acknowledging the full set of explicit and implicit variables that come into play when granting a specific clinical responsibility to a trainee [13], it is itself a linguistic articulation of a shift in thinking about assessment. Entrustment approaches to CBME translate competencies into sets of entrustable professional activities (EPAs) and ask medical educators to focus on the level of supervision they deem a learner needs to carry out such activities safely. The idea is that, instead of being determined solely in the abstract by rating learners against competency checklists, competence can (additionally) be inferred from these practically informed judgments that force assessors to take into account all that is necessary to make entrustment decisions. In effect, then, entrustment is a way of incorporating judgment in formal assessment, a means of addressing the tension between the need, on the one hand, to support fairness and shared understanding through published evaluation criteria that are applied in observation and documentation, *and*, on the other hand, the need to honor the frequently tacit expert knowledge at the heart of both medical and educational assessment practices. To what extent and in what ways it does so successfully remains to be seen, as current iterations of entrustment-based CBME are still relatively new. We argue that future inquiry into the varied ways in which entrustment is being implemented can teach us a good deal about the languaging^2^ of judgment and that such research should be used to enable sound and meaningful inclusion of judgment in formal assessment. Language used in assessment forms is not a matter of taste; it is a significant component of validity that steers response processes of raters [14, 15].

## Language in ad hoc and summative entrustment decisions

Despite the paucity of studies on the actual use of EPAs [16, 17], we focus on one key issue that has emerged so far in implementations of entrustment. This issue relates to the distinction made in the literature between ad hoc and summative iterations of entrustment and to their respective languaging in retrospective and prospective scales [13, 18]. Ad hoc entrustment happens every day in healthcare settings, largely as part of the flow of ordinary work-based teaching practices. The decisions to entrust here are based on a mix of estimated trustworthiness of the trainee, estimated risks in the particular situation, and the urgency of the task. In making an ad hoc entrustment, an attending is delegating *this task at this point in time*; there is no precedent being set. In North America, only ad hoc forms of entrustment are employed as part of formal assessment. However, in other places—the Netherlands, for instance—summative entrustments for EPAs are becoming the primary focus of assessment in postgraduate training. In summative entrustment, the decision of a clinical competency committee is that *from here on in* the resident is permitted to carry out this activity with no (or a specified level of) supervision. It is a decision that is not made in the moment but on the basis of evidence collected over time and from multiple sources, and it is ideally made collectively by people who have worked with the resident. Limited ratings are not enough. The distinction between ad hoc and summative entrustment focuses us on the points at which decisions are made about a learner’s need for supervision, and it is at these points that the requirement for documentation can stand in tension with recognition of assessors’ judgment.

Ad hoc and summative entrustment decisions are documented (i.e. “languaged”) using scales. The affordances and constraints of these linguistic artefacts used in entrustment, combined with their intended purposes and the parameters for their use, determine the extent to and ways in which judgment can legitimately be part of competency-based assessment.

## Retrospective scales

Ad hoc decisions, when they are recorded, appear in a retrospective scale, a scale that “looks back” and reports on how much supervision or help was provided or needed when a learner was entrusted with a task in one particular situation. It records observable facts (what the attending did) rather than impressions (what the attending felt). The Zwisch scale, used in surgery training [19], for example, records whether the attending engaged in “show and tell,” “active help,” “passive help,” or “supervision only.” It is incorporated in the US-based Procedural Learning and Safety Collaborative’s System for Improving and Measuring Procedural Learning (or SIMPL) app to facilitate resident intraoperative evaluation in every procedure in which they participate [20, 21]*.* The five-point Ottawa scale, also developed for surgery [22], similarly records what was done in the way of supervisory intervention. It is used in observation templates developed by the Royal College of Physicians and Surgeons of Canada (RCPSC) (see http://www.royalcollege.ca/rcsite/cbd/implementation/epa-examples-guides-e), and it runs from 1 (I had to do) to 5 (I did not need to be there). The assessor’s subjective judgment, woven into the flow of work-based training processes, is surfaced only briefly in textual form as an unelaborated tick mark. While such documentation usually occasions important and timely oral feedback for the learner, the main purpose of filling in and filing away (in, for example, a learner’s portfolio) a retrospective scale is to create data for a future decision about a learner’s formal advancement and their permitted or expected level of autonomy going forward.

In contexts in which only ad hoc forms of entrustment are practiced, it may be that retrospective scales are the primary (and perhaps only) means by which such judgment is operational in the assessment process. If the tick marks on these scales are meant to be compiled to draw out a pattern and that pattern is in effect believed to speak for itself, then there is no need for further expressions of (or deliberations around) trust-related judgment. Telling stories about one’s experiences with the resident, conveying one’s impressions of them, or discussing with others one’s feelings of confidence and/or uncertainty about a resident’s competence in a committee meeting—these not only become superfluous but risk inviting unfair bias and hearsay. Such considerations appear to factor into the position of the Canadian Royal College and other regulatory bodies in prohibiting competency committees from drawing on “previously undocumented” material in making decisions (Tam J, Wadhwa A, Martimianakis T, Fernando O, Regehr G. The role of previously undocumented data in the assessment of medical trainees in clinical competency committees. [article in preparation]). The algorithm of ad hoc entrustments, “objectively” recorded in terms of unelaborated supervisory actions, is then assumed to be an adequate and defensible way of incorporating assessors’ judgment into the body of evidence needed to justify advancement.^3^

Of course, such an assumption can be questioned. An ad hoc entrustment decision is, by definition, context-specific so that some further information is needed to determine what a tick mark indicates about the learner’s competence. It is conceivable, for instance, that “I had to do” had more to do with the fact that the operating room was running behind schedule than with a resident’s lack of skill. Conversely, “I did not need to be there” during a quiet night shift may be a hindsight rationalization rather than reflect the resident’s mastery. Some additional detail might be added to the form. Instead of simply checking off “I had to be there just in case,” an attending might render things more clear by adding, for example, “Injection on patient was completed safely but not smoothly. Patient was squeezing eye during eyelid speculum placement and was not still during injection itself.” Still, whether this is adequate is open to question. The complexity of the case is obviously a key factor in ad hoc entrustment (and one both the American SIMPL app and the Canadian Royal College’s templates record), but there will be others at play, too, such as how much experience the resident had up to that point, how busy the clinic was that day, what other healthcare providers (e.g., nurses) remained with the resident, and so on. How much additional information, then, needs to be documented together with retrospective scales in ad hoc entrustment? And will it consist only of easily documentable, “neutral” factors like the aforementioned, or will more “personal” and less readily recordable information also be included (anything from an acknowledgement of problems a resident is dealing with in their private life that may contribute to an unusually suboptimal performance, to a reflection by the assessor on personal practice preferences [23] that might enter into a decision to entrust or not)? It must be remembered that assessors may not just find certain kinds of knowledge challenging to surface in language, but that they may also be reticent to document information perceived as sensitive in official forms that become part of an institutional record. Communicating information to contextualize a judgment is not just a matter of documenting neutral facts; it affects relationships and may have consequences that are unintended. The moment the ad hoc data are compiled in support of a more comprehensive, future-oriented decision about advancement, considerations about how much and what kinds of additional information to include become pressing issues, for how is this compilation to be interpreted? Unless the “muddy” nature of the unelaborated data points used to build the algorithm is ignored, it must be accepted that more knowledge is needed and that some of that knowledge will not be easily (or willingly) documented.

## Prospective scales

Summative entrustment decisions are typically recorded using a prospective scale. As the name suggests, these are forward looking, pronouncing on the level of supervision a learner requires to carry out a particular EPA in future. The five-point generic scale described by Ten Cate & Scheele [24] is an example of a prospective scale.^4^ As used by competency committees, it enacts, rather than reports on, entrustment—it is the equivalent of a *performative*, an utterance in which the deed is accomplished in the saying or writing. While all language use may be considered a form of action and while retrospective scales clearly play a role in the act of advancing learners, we are here referring to Austin’s [25] early distinction between *constatives*, utterances that report on some reality and are judged according to whether they are true or false, and *performative*s, utterances which act and are not truth-evaluable but, rather, in Austin’s terms, “happy” or “unhappy”—in effect, successful or not in accomplishing what was intended. It is this real-world orientation that generates risky but also, proponents would argue, more meaningful assessments.

A summative entrustment of an EPA entails risk in a way that an ad hoc entrustment does not, of course. In ad hoc entrustments there is not only closer oversight, but subsequent written declarations that the learners could have performed the observed procedure on their own relate to a particular, already completed performance, not to any number of performances in unpredictable future cases. The truthfulness of a declaration could be contested by someone who evaluates the learner’s performance differently, but such disagreement would be unlikely to produce negative consequences of any real significance. A summative entrustment, by contrast, involves relinquishing oversight, and giving authorization to a learner into the future involves not only greater uncertainty about consequences for patient safety, but also, perhaps, uncertainty about other real-world consequences such as professional reputation and legal responsibility if things don’t go well. Smit et al.[26] report that EPAs obtained by learners in the Netherlands are not always readily acknowledged by other units, departments, or institutions, and that the possibility of legal challenges remain a concern. This suggests that the performative success of pronouncements of summative entrustment is still ambiguous.

Summative entrustment, then, may prompt deeper and more cautious introspection. Documented evidence will certainly be important; among the items that are mandated by electronic portfolio assessment systems used in Dutch university medical centers are mini-Clinical Evaluation Exercise (CEX) feedback, Critical Appraisal of a Topic (CAT), case-based discussions, multi-source feedback, rotation reviews, progress reports, evidence of completed training courses, test results, and prior entrustment decisions. However, the act of stating officially that only ‘distant supervision is henceforth required’ will likely call forth a more conscious appeal to one’s own judgment, as well, and to the experiences and impressions of colleagues who have also worked with the learner. For proponents of summative entrustment decisions during training (not only at the very end, through licensing and certification), risk is thus an inevitable but also valuable part of medical education. In combining evaluation of competence with an assessment of risk, summative entrustment moves the assessor beyond merely observing and reporting a learner’s performance, and forces them to think very deliberately (and in very personal terms) about what is at stake when according a learner responsibility at a given level of supervision [18].

The question remains as to how to maximize transparency when drawing upon judgment in this way. Again, we need to ask how much and what types of documented information needs to accompany these high-risk decisions (and how it is to be used). This question gains urgency when the previously accumulated documentary evidence does not readily point to a decision one way or the other, or when it points in a direction that does not align with assessors’ experiences, impressions, or “gut feelings.” To what extent do we require (documented) explication or justification of a summative decision by a competency committee and to what extent can we, in effect, just trust the entrusters’ judgments on the basis of their expertise? Operational guidelines for competency committees tasked with summative entrustment decisions are not widely documented. Smit et al.’s [26] report on a Dutch EPA-based graduate pediatrics program offers some clues as to what a systematized process might look like, including the use of an electronic form that all supervisors fill out and submit prior to a competency committee meeting and that serves as the basis for discussions about similarities and differences among the supervisors’ independent assessments. Such discussions, to which residents are not party, are required to result in consensus. While Smit et al. report that the structured nature of the decision process is felt by users to contribute to robust decisions and high-quality feedback to learners, there are no formal guidelines regarding the documentation and filing of the committee’s deliberations (R. Gemke, 12 December 2019, email to authors).

## Adapting the language for implementing entrustment

The languaging of entrustment has received relatively limited attention in the literature so far, but there are indications that, in moving ahead, medical educators will need to adapt the linguistic artefacts used to implement it. Thus, the scales may have to be made more amenable to the requirements of different specialties and different contexts. Hatala et al. [27] note that the Ottawa scale’s focus on the need to “be there” is an ill fit for specialties like internal medicine, where supervision is frequently indirect and where the system itself often ends up “entrusting” residents with responsibilities. However, the same scale might sit awkwardly with anesthesia supervisors in Canada and the US precisely because it includes the option “didn’t need to be there” when, by law, they are required to be present during a case (P. Tanaka, 1 and 12 December 2019, emails to the authors; L. Bosma, 23 October 2019, personal communication). This concern would not arise in New Zealand and Australia or in Switzerland, where trainees may be entrusted with sole anesthesia care with the supervisor remaining at home (J. Weller, 1 December 2019, email to the authors; A. Marty, 1 December 2019, email to the authors; see also Weller et al. [28]).

As entrustment continues to be implemented in assessment in health professions education, we should research and refine the linguistic artefacts used, and we should attend not only questions of how well the language fits specialty-specific and context-specific needs but also to the more general question of how well it allows judgment to play a legitimate role. How well do the artefacts through which entrustment is implemented manage the tension between the often-tacit nature of judgment and the desire for transparency in assessment?

At present, entrustment is documented either retrospectively—ad hoc decisions languaged briefly as data points so as not to disrupt the flow of work-based training—or prospectively—summative decisions languaged as formal enactments of entrustment, predicated on prior evidence and collective deliberation. In both cases the scales used are linguistically spare and so raise questions about how much and what kinds of explicitness should accompany them. If we acknowledge that tacit judgments are a strength in formal assessment, then we need to better understand how they can be harnessed meaningfully and responsibly by the linguistic artefacts (and related practices) we employ to support fairness and accountability^5^. We need to ensure those artefacts contribute to good assessment, not through the strict policing of judgment such that it risks being quelled, but as tools for organizing assessors’ thoughts and feelings and for negotiating shared ground among stakeholders.

## Final thoughts: Trusting the truster

If it is accepted that high quality assessment should include implicit expert judgment, then medical education will have to let go of the expectation that all assessment can and should be fully and at all times explainable, let alone documentable. Demands for transparency should not come at the expense of knowledge that is not easily expressible in words (and certainly not always moldable into predefined categories of a proficiency checklist) but that is nonetheless key to determining a learner’s readiness for entrustment with a critical task or, indeed, with a license to practice, for that is the ultimate task of assessment. If such knowledge is disregarded, the quality of entrustment decisions may suffer. A transcript of scores, a checklist of achievements, or the knowledge that a trainee has spent the required number of months or years in training are, at best, limited justifications for that trust. When it comes to entrustment decisions, then, whether ad hoc by individual supervisors or summative around a committee table, a climate of “trust in trusters” must supplement the available “objective” information.

Trust involves the acceptance of risk. Trusting medical trainees to work by themselves, either deliberately during training for distinct EPAs or at the moment of licensing or specialty certification for the breadth of a whole profession, expresses the educator’s or the institution’s acceptance of associated risks. Ultimately there are no guarantors of competence, only evidence that can inform the risk taken in declaring someone competent. Expert judgment is certainly not infallible as evidence, and experts differ in their judgments. However, to limit evidence in assessment only to knowledge that can be fully and formally languaged would be naïve and would impoverish assessment.

## Footnotes

1 While this paper focuses on whether and how language can mitigate the risks of unfairness in assessment without eradicating valuable, but often tacit, impressions and gut feelings, it is important to stress from the outset that explicitness in no way guarantees fairness. Critical linguistic analysis has long drawn attention to racial and gender biases existing in text, and the texts employed in the assessment of medical trainees are no exception. For example, a study [29] of 1300 narrative comments from direct observation evaluations revealed qualitative differences in the kind of feedback that male and female emergency medicine residents received from attending physicians.

2 We use this transitive verb to mean “to express in language”, i.e., to bring into a system of communication that enables the socially meaningful exchange of verbal or symbolic utterances.

3 The Canadian Royal College templates do include space for recording narrative comments. What types of comments are written and how these are used by competency committees in conjunction with data from the scales remains to be seen.

4 The generic scale runs as follows: 1‑Presence allowed but no permission to enact EPA; 2‑Practice EPA with direct (pro-active) supervision allowed; 3‑Practice EPA with indirect (re-active) supervision allowed; 4‑Unsupervised practice for this EPA allowed (distant oversight); 5‑May provide supervision to junior learners for this EPA.

5 While this article focuses on judgment in assessment, it may also play a valuable role in enhancing feedback to learners. Here, too, a focus on language will be important: How does one share one’s impressions and “gut feelings” with learners such that they can learn from them?.

## References

[1] Govaerts M, Van Der Vleuten CPM (2013). Validity in work-based assessment: expanding our horizons. Med Educ.

[2] Hodges B (2013). Assessment in the post-psychometric era: learning to love the subjective and collective. Med Teach.

[3] Gingerich A (2015). What if the ‘trust’ in entrustable were a social judgement?. Med Educ.

[4] ten Cate O, Regehr G (2019). The power of subjectivity in the assessment of medical trainees. Acad Med.

[5] Colbert CY, French JC, Herring ME, Dannefer EF (2017). Fairness: the hidden challenge for competency-based postgraduate medical education programs. Perspect Med Educ.

[6] Powell D, Carraccio C (2018). Toward competency-based medical education. N Engl J Med.

[7] Frank JR, Snell LS, Ten Cate O, Holmboe ES, Carraccio C, Swing SR (2010). Competency-based medical education: theory to practice. Med Teach.

[8] Lockyer J, Carraccio C, Chan MK (2017). Core principles of assessment in competency-based medical education. Med Teach.

[9] Sagasser MH, Fluit CRMG, Van Weel C, Van Der Vleuten CPM, Kramer AWM (2017). How entrustment is informed by holistic judgments across time in a family medicine residency program: an ethnographic nonparticipant observational tudy. Acad Med.

[10] Arnal SG, Burwood S (2003). Tacit knowledge and public accounts. J Philos Educ.

[11] Alofs L, Huiskes J, Heineman MJ, Buis C (2015). User reception of a simple online multisource feedback tool for residents. Perspect Med Educ.

[12] Buis CAM, Eckenhausen MAW, Ten Cate O (2018). Processing multisource feedback during residency under the guidance of a non-medical coach. Int J Med Educ.

[13] ten Cate O, Hart D, Ankel F (2016). Entrustment decision making in clinical training. Acad Med.

[14] Plake B, Wise L (2014). AERA, APA, NCME. Standards for educational and psychological testing. revised.

[15] Gingerich A, Ramlo SE, van der Vleuten CPM, Eva KW, Regehr G (2017). Inter-rater variability as mutual disagreement: identifying raters’ divergent points of view. Adv Health Sci Educ.

[16] O’Dowd E, Lydon S, O’Connor P, Madden C, Byrne D (2019). A systematic review of 7 years of research on entrustable professional activities in graduate medical education, 2011–2018. Med Educ.

[17] Hauer KE (2019). Seeking trust in entrustment: shifting from the planning of entrustable professional activities to implementation. Med Educ.

[18] ten Cate O, Schwartz AJ, Chen HC (2020). Assessing trainees and making entrustment decisions: on the nature and use of entrustment and supervision scales. Acad Med.

[19] George BC, Teitelbaum EN, Meyerson SL (2014). Reliability, validity, and feasibility of the Zwisch Scale for the assessment of intraoperative performance. J Surg Educ.

[20] Bohnen JD, George BC, Williams RG (2016). The feasibility of real-time intraoperative performance assessment with SIMPL (System for Improving and Measuring Procedural Learning): early experience from a multi-institutional trial. J Surg Educ.

[21] George BC, Bohnen JD, Schuller MC, Fryer JP (2019). Using smartphones for trainee performance assessment: a SIMPL case study. Surgery.

[22] Gofton WT, Dudek NL, Wood TJ, Balaa F, Hamstra SJ (2012). The Ottawa Surgical Competency Operating Room Evaluation (O-SCORE): a tool to assess surgical competence. Acad Med.

[23] Apramian T, Cristancho S, Sener A, Lingard L (2018). How do thresholds of principle and preference influence surgeon assessments of learner performance?. Ann Surg.

[24] Ten Cate O, Scheele F (2007). Competency-based postgraduate training: can we bridge the gap between theory and clinical practice?. Acad Med.

[25] Austin JL (1975). How to do things with words.

[26] Smit MP, de Hoog M, Brackel H, Ten Cate O, Gemke R (2019). A national process to enhance the validity of entrustment decisions for Dutch pediatric residents. J Grad Med Educ.

[27] Hatala R, Ginsburg S, Hauer KE, Gingerich A (2019). Entrustment ratings in internal medicine training: capturing meaningful supervision decisions or just another rating?. J Gen Intern Med.

[28] Weller JM, Misur M, Nicolson S (2014). Can I leave the theatre? a key to more reliable workplace-based assessment. Br J Anaesth.

[29] Mueller AS, Jenkins TM, Osborne M, Dayal A, O’Connor DM, Arora VM (2017). Gender differences in attending physicians’ feedback to residents: a qualitative analysis. J Grad Med Educ.

